# Brassinosteroids regulate pavement cell growth by mediating BIN2-induced microtubule stabilization

**DOI:** 10.1093/jxb/erx467

**Published:** 2018-01-10

**Authors:** Xiaolei Liu, Qin Yang, Yuan Wang, Linhai Wang, Ying Fu, Xuelu Wang

**Affiliations:** 1 Shanghai Center for Plant Stress Biology, Chinese Academy of Sciences, Shanghai, China; 2 State Key Laboratory of Genetic Engineering and Institute of Plant Biology, School of Life Sciences, Fudan University, Shanghai, China; 3 State Key Laboratory of Plant Physiology and Biochemistry, Department of Plant Sciences, College of Biological Sciences, China Agricultural University, Beijing, China; 4 College of Life Science and Technology, Huazhong Agricultural University, Wuhan, China; 5 Department of Botany and Plant Science, University of California Riverside, Riverside, CA, USA

**Keywords:** BIN2, brassinosteroids, cytoskeleton, microtubule, pavement cell, tubulin protein

## Abstract

Brassinosteroids (BRs), a group of plant steroid hormones, play important roles in regulating plant development. The cytoskeleton also affects key developmental processes and a deficiency in BR biosynthesis or signaling leads to abnormal phenotypes similar to those of microtubule-defective mutants. However, how BRs regulate microtubule and cell morphology remains unknown. Here, using liquid chromatography–tandem mass spectrometry, we identified tubulin proteins that interact with Arabidopsis BRASSINOSTEROID INSENSITIVE2 (BIN2), a negative regulator of BR responses in plants. *In vitro* and *in vivo* pull-down assays confirmed that BIN2 interacts with tubulin proteins. High-speed co-sedimentation assays demonstrated that BIN2 also binds microtubules. The Arabidopsis genome also encodes two BIN2 homologs, BIN2-LIKE 1 (BIL1) and BIL2, which function redundantly with BIN2. In the *bin2-3 bil1 bil2* triple mutant, cortical microtubules were more sensitive to treatment with the microtubule-disrupting drug oryzalin than in wild-type, whereas in the BIN2 gain-of-function mutant *bin2-1*, cortical microtubules were insensitive to oryzalin treatment. These results provide important insight into how BR regulates plant pavement cell and leaf growth by mediating the stabilization of microtubules by BIN2.

## Teaser

The stabilization of microtubules by interaction with BRASSINOSTEROID INSENSITIVE2 (BIN2), a negative regulator of brassinosteroid responses, links brassinosteroid signaling to cytoskeletal dynamics and the regulation of plant development.

## Introduction

Brassinosteroids (BRs) are a group of steroid hormones that play important roles in regulating plant development, including vascular differentiation, senescence, stress responses, and photomorphogenesis (Clouse *et al.*, 1996; Li and Chory, 1997). A deficiency in BR biosynthesis or signaling leads to dwarf phenotypes due to retarded plant growth (Li and Chory, 1997; Clouse and Sasse, 1998). BR regulates plant cell and organ growth by stimulating transverse microtubule organization (Catterou *et al.*, 2001).

Many components of the BR signaling pathway have been identified. BR is perceived by the transmembrane serine/threonine protein kinase BRASSINOSTEROID INSENSITIVE 1 (BRI1) (Li and Chory, 1997). The *bri1* mutant is morphologically similar to BR biosynthetic mutants and is insensitive to exogenous BR application (Clouse *et al.*, 1996; Li and Chory, 1997; Noguchi *et al.*, 1999; Friedrichsen *et al.*, 2000). BR binds to the extracellular domain of BRI1 and induces heterodimerization of BRI1 and BAK1, leading to their activation (Li *et al.*, 2002; Nam and Li, 2002; Santiago *et al.*, 2013). The binding of BRI1 to BAK1 is negatively regulated by BRI1 KINASE INHIBITOR1 (BKI1), a plasma membrane-associated protein (Wang and Chory, 2006). In the absence of BR, BKI1 binds to the kinase domain of BRI1 and prevents the interaction between BAK1 and BRI1 (Wang and Chory, 2006). The activation of BRI1 leads to the dissociation of BKI1 from the plasma membrane into the cytoplasm (Wang and Chory, 2006). BIN2, one of the ten glycogen synthase kinase 3 (GSK3)-like kinases in Arabidopsis, is another negative regulator of BR responses (Li *et al.*, 2001; Li and Nam, 2002). *BIN2* encodes a 380 aa protein that contains an N-terminal variable domain, a conserved kinase domain and a C-terminal domain. A highly conserved four-amino-acid TREE motif (threonine–arginine–glutamic acid–glutamic acid) in the C terminus is important for the activity of BIN2, and a mutation in the TREE motif affected the degradation of BIN2. In the absence of BR, BIN2 phosphorylates BRASSINAZOLE-RESISTANT1 (BZR1) and BRI1-EMS-SUPPRESSOR1 (BES1), which are targeted for degradation by the proteasome (Yin *et al.*, 2002, 2005).

The cytoskeleton plays important roles in regulating cell morphogenesis, intracellular transport, and cell division. Microtubules are formed by the polymerization of α- and β-tubulin dimers. The intrinsic polarity of microtubules provides them with a less-dynamic minus end and a more-dynamic plus end. During polymerization, tubulin molecules are positioned within a microtubule so that α-tubulin faces the slow-growing minus end and β-tubulin faces the fast-growing plus end. The dynamic instability of the cytoskeleton allows it to quickly alter plant growth and development to help the plant adapt to various conditions. Loss-of-function mutants of major microtubule components usually exhibit abnormal growth (Nakajima *et al.*, 2004), and altering the dynamic instability of microtubules leads to right-handed helical growth (Abe and Hashimoto, 2005). For instance, the *pilz* mutant fails to form basic microtubule structures, and its growth is arrested at early stages of embryogenesis (Mayer *et al.*, 1999). The *fass* mutant, which is unable to correctly form microtubule structures, shows defects in polarized cell growth and radial pattering (Torres-Ruiz and Jürgens, 1994). Plants carrying mutations in *SPIRAL1* (*SPR1*) and *SPR2*, which participate in the organization of microtubule structure, show increased radial growth and shorter cells compared with wild-type (Furutani *et al.*, 2000). Disrupting the microtubules results in abnormal cell shape and plant development. However, little is known about how cytoskeletal dynamics are regulated, although auxin was shown to induce the reorganization (Takahashi *et al.*, 1995) and depolymerization of microtubules (Baluska *et al.*, 1996).

The BR receptor mutant *bri1* has round leaves, short petioles, and reduced apical dominance. *BRI1*-overexpressing plants exhibit right-handed helical growth, and *bzr1-1D* and *bes1-D* mutants have curly hypocotyls and leaves (Wang *et al.*, 2002). The expression of *TUB1* is affected in the BR-deficient mutant *dim* (Takahashi *et al.*, 1995; Klahre *et al.*, 1998). Another BR-defective mutant, *bul1-1* (*boule 1-1*), exhibits reduced microtubule levels and a total lack of parallel microtubule organization, which can be rescued by BR treatment (Catterou *et al.*, 2001). The similarity in phenotype between BR and cytoskeleton mutants and the ability of BR to rescue microtubule organization suggest that BR might regulate plant development by regulating cytoskeletal dynamics (Catterou *et al.*, 2001). A recent study showed that BZR1 targets and up-regulates *MDP40* (*MICROTUBULE DESTABILIZING PROTEIN 40*) to mediate hypocotyl cell elongation by influencing the orientation and stability of cortical microtubules (Wang *et al.*, 2012). Brassinolide (BL, an active BR) treatment also alters the orientation of microtubules in hypocotyls and increases the sensitivity of microtubules to the microtubule-disrupting drug oryzalin by regulating the expression of *MDP40*. Although BR was shown to regulate plant development and hypocotyl elongation through regulating microtubule organization, little is known about how defective BR signaling leads to morphological defects in the leaves of these mutants due to abnormal formation of the cytoskeleton. While the process of leaf pavement cell development is more intricate than that of hypocotyls, whether the mechanism underlying BR-regulated microtubules in pavement cells is the same as that in hypocotyls remains to be investigated.

Microtubule dynamics are regulated by the acetylation, tyrosination, detyrosination, polyglutamylation, polyglycylation, palmitoylation, and phosphorylation of tubulins (LeDizet and Piperno, 1987; Eddé *et al.*, 1991). Among these, protein phosphorylation is the most common and important post-translational modification. Protein phosphorylation plays important roles in the dynamic instability of microtubules. For instance, the phosphorylation of Ser172 in β-tubulin by cyclin-dependent kinase (Cdk1) can impair both its binding to GTP and the interaction between tubulin dimers (Fourest-Lieuvin *et al.*, 2006). Furthermore, CASEIN KINASE 1-LIKE 6 (CKL6), which contains a tubulin-binding domain, plays a role in anisotropic cell growth and shape formation in Arabidopsis by regulating microtubule organization, possibly through the phosphorylation of tubulins (Ben-Nissan *et al.*, 2008). A kinase-inactive mutant form of CKL6 induces altered cortical microtubule organization and anisotropic cell expansion, and calmodulin-dependent kinase can also phosphorylate tubulins and microtubule-associated proteins (Goldenring *et al.*, 1983; Ben-Nissan *et al.*, 2008).

In this study, we searched for BIN2-interacting proteins by liquid chromatography–tandem mass spectrometry (LC-MS/MS) of immunoprecipitated (IPed) proteins from BIN2-FLAG transgenic plants, finding that BIN2 interacted with all tubulin proteins. Whereas microtubule-associated mutants display an isotropically expanding cell phenotype, including distorted trichomes, swollen roots, short hypocotyls, and aberrant pavement cells (Mathur, 2004), the BR biosynthesis mutant *det2* (*deetiolated 2*), the BR signaling-defective mutants *bri1-5* and *bin2-1*, and a *BKI1*-overexpression line (*BKI1-OX*) all showed short hypocotyls, round leaf shapes, and abnormal pavement cells. We therefore reasoned that BR regulates pavement cell development through its effect on microtubules. We further confirmed that BIN2 interacts with tubulin proteins both *in vitro* and *in vivo*. Co-sedimentation assays indicated that BIN2 directly binds to microtubules. Treatment with the microtubule-disrupting drug oryzalin confirmed that BIN2 indeed inhibits microtubule disruption. Our findings suggest that BR regulates plant development and pavement cell development by mediating microtubule stabilization induced by BIN2.

## Materials and methods

### Plant materials and growth conditions

Arabidopsis plants, including wild-type (Col-0 and Ws-2), *det2* (en2), *bin2-1* (Col-0), the *bin2-3 bil1 bil2* triple mutant (Ws-2), *bri1-5* (Ws-2), *BKI1* overexpression lines (Col-0), BRI1 overexpression lines (Col-0), sav2 (Col-0), and *bes1-D* (Col-0) were used in this study. Transgenic Arabidopsis plants expressing green fluorescent protein (GFP)-tagged α-tubulin crossed with BR mutants were used for observation of cortical microtubules. Seeds were grown on half-strength Murashige and Skoog (½MS) medium (pH 5.8) with 0.8% agar and 1% sucrose for 7 d at 22 °C. The plants were grown at 22 °C under a 16 h light–8 h dark photoperiod.

### Microscopy and image analysis

Samples were imaged using a Leica TCS SP8 laser scanning confocal microscope and Spinning disk confocal microscope. Images were obtained with ×25 objective for propidium iodide (PI) staining and ×40 and ×60 objective for microtubule labelling. For the microtubule labelling, maximum Z-projections were used and the Z-step size was 1 μm. Images were captured by a Leica TCS SP8 at 488 and 561 nm laser excitation and 500–550 nm and 590–630 nm emission for GFP and PI staining. Images captured by a spinning disk confocal microscope were obtained with ×40 and ×60 objective. For the microtubule labelling, maximum Z-projections were used and the Z-step size was 1 μm. Images were captured at 488 nm laser excitation and 500–550 nm emission.

PI (5 μg ml^−1^)-stained cotyledon pavement cells from 7-day-old seedlings were imaged. ImageJ software was used to measure the lobe number, lobe length, neck width, perimeter, and area (http://rsb.info.nih.gov/ij). Circularity was analysed according to Zhang *et al.* (2011).

Microtubule alignment was quantified according to Gomez *et al.* (2016). At least 50 cells were used. The values were recorded and the significance was analysed using Student’s paired *t*-test.

### BRZ, eBL, and bikinin treatment

For the BRZ (224047-41-0, Cayman Chemical), eBL (78821-43-9, Sigma-Aldrich), and bikinin (188011-69-1, Sigma-Aldrich) treatment assays, wild-type seeds were grown on ½MS medium with 1 μM BRZ, 1 μM eBL, or 30 μM bikinin for 7 d, followed by observation of pavement cells in the seedlings.

### Plasmid construction and recombinant protein purification

For the glutathione *S*-transferase (GST) pull-down assays, *BIN2*, *bin2-1*, and *BIN2*^*K69R*^ were cloned into the pGEX4T-1 vector, and *TUBULIN* genes were cloned into the pET28a vector. BIN2–GST, bin2-1–GST, BIN2^K69R^–GST, and TUA1–His, TUA3–His, TUA5–His, TUB3–His, TUB4–His, TUB6–His, TUB7–His, and TUB8–His were expressed in *Escherichia coli* cells (Rosetta strain) and purified using glutathione resin and Ni-NTA agarose.

### Protein extraction from plants

Plants were ground to a fine powder in liquid nitrogen, solubilized with 2× extraction buffer (100 mM Tris–HCl pH 7.5, 300 mM NaCl, 1% Triton X-100, 10% glycerol, and protease inhibitor), separated on a 10% SDS-PAGE gel, transferred to a nitrocellulose membrane (Amersham Biosciences), and then detected with corresponding antibodies.

### Pull-down and semi-*in vivo* pull-down assays

Purified proteins, including GST, BIN2–GST, BIN2^K69R^–GST, and bin2-1–GST were bound to 20 μl GST resin in binding buffer (1× PBS, pH 7.4, 0.1% Triton X-100) for 2 h at 4 °C. The beads were washed three times and incubated with purified tubulin-His proteins for 2 h at 4 °C for binding. Binding buffer (80 μl) and 20 μl 5× SDS loading buffer were added to the samples, which were boiled for 5 min and separated by 10% SDS-PAGE. His antibody (CWBIO, CW0286, monoclonal) and α-tubulin antibody (Beyotime, AT819, monoclonal) were used for detection, goat anti-mouse horseradish peroxidase (HRP)-conjugated secondary antibodies (CWBIO, CW0145) were used. An ECL Substrate Kit (Thermo Fisher Scientific, NCI4106) was used to detect the HRP-conjugated secondary antibodies. After incubated with ECL buffer (A:B, 1:1) for 2 min at room temperature, the polyvinylidene fluoride membrane was put into a cassette and the signals were visualized with X-ray film.

For the semi-*in vivo* pull-down assay, GST and BIN2–GST, BIN2^K69R^–GST, and bin2-1–GST were bound to 20 μl GST resin in binding buffer (1× PBS, pH 7.4, 0.1% Triton X-100) for 2 h at 4 °C. GST was used as a negative control. After three washes, tubulin proteins purified from porcine brain were added to the samples, followed by rebinding for 2 h at 4 °C. Tubulin antibody was used for detection.

Plants overexpressing TUA6-GFP were ground with liquid nitrogen and total protein was extracted with 2× extraction buffer (100 mM Tris–HCl pH 7.5, 300 mM NaCl, 1% Triton X-100, 10% glycerol, and protease inhibitor). Protein extracts were centrifuged at 20000 *g* for 10 min, and the resulting supernatant was incubated with prewashed BIN2–GST beads for 1 h at 4 °C. Beads were resuspended with 1× SDS-PAGE loading buffer and analysed by SDS-PAGE and immunoblotting.

### *In vitro* kinase assay

For the phosphorylation assay, BIN2–GST or BIN2^K69R^–GST and tubulins from porcine brain were incubated in 24 μl reaction buffer (50 mM HEPES pH 7.4, 10 mM MgCl_2_, 10 mM MnCl_2_, 1 mM DTT, 10 μM ATP, 1 μl [10 μCi] [γ-^32^P]ATP) for 1 h at 37 °C. The reaction was terminated by adding 6 μl 6×SDS loading buffer. The samples were separated by 10% SDS-PAGE and stained with Coomassie Brilliant Blue, followed by drying and autoradiography of the gel.

### Microtubule binding assay

GST–BIN2, GST–BIN2^K69R^, and GST–bin2-1 were purified and protein concentrations determined using a Bio-Rad protein assay kit. Porcine brain tubulins were purified as described previously (Castoldi and Popov, 2003). The microtubule polymerization and co-sedimentation experiments were performed as previously described (Mao *et al.*, 2005).

Recombinant GST–BIN2, GST–BIN2^K69R^, and GST–bin2-1 were co-sedimented with 5 mM Taxol-stabilized microtubules in PEMT buffer (100 mM PIPES, 1 mM EGTA, 1 mM MgCl_2_, and 20 μM Taxol, pH 6.9). After incubation at 25 °C for 30 min, the samples were centrifuged at 100000 *g* for 30 min at 25 °C. Pellets and supernatants were analysed by 10% SDS-PAGE and visualized by staining with Coomassie Brilliant Blue R250. GST was used as a negative control.

### Oryzalin treatment

Cotyledon pavement cells from 7-day-old seedlings including wild-type, *bin2-3 bil1 bil2*, and *bin2-1* with GFP–α-tubulin expressed in the background were used for observation. Oryzalin (3,5-dinitro-*N*^4^,*N*^4^-dipropylsulfanilamide, Ps-410, 19044-88-3, Sigma-Aldrich) was applied to whole plants at the indicated concentrations. Seedlings were treated with 15 μM oryzalin in tubes for 3 and 10 min, and then the seedlings were put on a glass slide and observed using confocal microscopy.

### Real-time PCR analysis

Total RNA was extracted from seedlings using the Qiagen RNAprep plant kit. Primers for real-time PCR are listed in Supplementary Table S1 at *JXB* online. The *UBOX* gene was used as a control to normalize the level of total RNA. PCRs were performed with a Bio-Rad CFX96 real-time system.

## Results

### BR regulates microtubule organization and pavement cell development

**Fig. 1.**
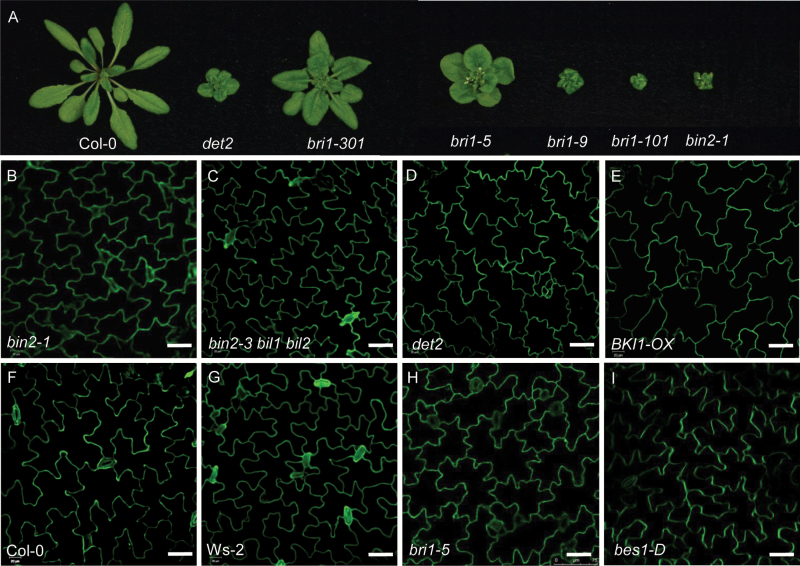
BR mutants showed abnormal pavement cell shapes. (A) Phenotype of BR-related mutants. (B–I) Pavement cell shape of *bin2-1* (B), *bin2 bil1 bil2* triple mutant (C), *det2* (D), *BKI1-OX* (E), Col-0 (F), Ws-2 (G), *bri1-5* (H), and *bes1-D* (I). Cotyledons stained by propidium iodide. Bars in (B–I): 40 μm.

BR biosynthesis and signaling-deficient mutants have rounder leaves and shorter petioles than wild-type (Fig. 1A). To investigate the cellular mechanism underlying these phenotypes, we examined pavement cell shape in the BR mutants using propidium iodide (PI) staining and confocal microscopy. Wild-type mature pavement cells exhibited a ‘jigsaw puzzle’ shape, with lobes interdigitated with the necks of neighboring cells (Fig. 1F). By contrast, the BR mutants exhibited a smaller area of pavement cells and defective lobe formation (Fig. 1B–I). Quantitative analysis (Table 1) confirmed that pavement cell area, perimeter, and circularity value were smaller, with wider necks, in BR signaling and biosynthesis-deficient mutants (such as *det2*, *bri1-5*, *bin2-1*, and *BKI1-Overexpression line* (*BKI1-OX*)) compared with wild-type. By contrast, in BR signaling overexpression lines such as *bes1-D*, there were more lobes with pointed tips and the lobes were fewer in number. In the *bin2-3 bil1 bil2* triple mutant, the area of the pavement cells was larger than in wild-type, with more lobes that were shorter in length. All tubulin-related mutants showed increased cell expansion, a phenotype similar to that of BR-related mutants such as *bri1*, *BKI1-OX*, *det2*, and *bin2-1*. Since the T-DNA insertion β-tubulin mutant *tub1* (SALK_036755C) and the α-tubulin mutant *tua6* (SALK_091096C) we ordered from the Arabidopsis Biological Resource Center (ABRC) are homozygous lethal, we used the β-tubulin mutant *tub4* (also designated *sav2*) for analysis; *sav2* was identified through a forward genetic screen and contains a point mutation in *TUB4* (Yu *et al.*, 2015). As shown in Supplementary Fig. S1, the *sav2* mutant had short petioles and round leaves, like BR mutants. PI staining showed that *sav2* pavement cells were also round, with wide necks (Supplementary Fig. S1). Quantitative analysis (Table 1) confirmed the phenotype of *sav2* is similar to BR mutants.

**Table 1.** The size of cotyledon pavement cells of BR mutants

	Background	Lobe no.	Lobe length (μm)	Neck width (μm)	Perimeter (μm)	Area (μm^2^)	Circularity
Col-0		9.1 ± 0.2	28.1 ± 4.8	31.2 ± 1.8	493.2 ± 14	7146 ± 382	0.37 ± 0.02
WS-2		10.1 ± 0.8	25.27 ± 0.2	25.20 ± 1.2	555 ± 11.3	5685 ± 324	0.23 ± 0.01
*BKI1-OX*	Col-0	13.3 ± 1.1****	14.2 ± 2.1****	35.1 ± 3.1****	381.4 ± 0.7****	4383 ± 269****	0.38 ± 0.02***
*BRI1-OX*	Col-0	8.6 ± 0.7****	17.0 ± 1.6****	27.6 ± 0.4****	369.9 ± 4.4****	4657 ± 218****	0.45 ± 0.02****
*det2*	En2	8.5 ± 0.4****	11.7 ± 2.0****	37.01 ± 3.0****	358.3 ± 8.8****	5418 ± 98****	0.53 ± 0.01****
*bin2-1*	Col-0	7 ± 0.4****	12.8 ± 1.2****	27.65 ± 1.3****	328 ± 13.5****	3334 ± 174****	0.39 ± 0.02****
*bin2-3 bil1 bil2*	WS-2	14.5 ± 2****	16.5 ± 1.7****	22.71 ± 2.6****	502.1 ± 8.6****	5704 ± 215****	0.28 ± 0.01****
*bri1-5*	WS-2	12 ± 1.3****	14.3 ± 1.1****	29.5 ± 2.3****	398.9 ± 16.6****	5495 ± 266****	0.44 ± 0.02****
*bes1-D*	En2	8.4 ± 0.37****	12.6 ± 1.1****	22.87 ± 2.2****	402.5 ± 7.8****	4920 ± 153****	0.38 ± 0.05***
*sav2*	Col-0	9.4 ± 0.8****	13.6 ± 0.13****	40.5 ± 3****	334 ± 81****	6278 ± 294****	0.71 ± 0.03****

*n*>63 for the lobe number, lobe length, and neck width data, and *n*>30 for the perimeter, area, and circularity measurements. The measurements were performed using ImageJ software (http://rsb.info.nih.gov/ij). *t*-Test: ***P<0.001, ****P<0.0001.

**Fig. 2.**
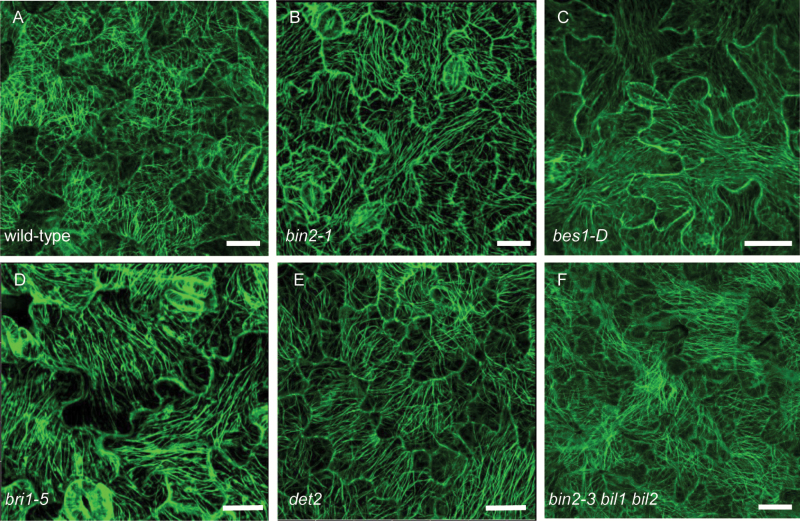
BR mutants showed parallel arrangement of microtubules. (A–F) Cortical microtubules of cotyledon pavement cells in wild-type (A), *bin2-1* (B), *bes1-D* (C), *bri1-5* (D), *det2* (E), and *bin2-3 bil1 bil2* triple mutant (F) with a transgene expressing GFP–tubulin in the background for all lines. Bars: 20 μm. (This figure is available in color at *JXB* online.)

The abnormal pavement cell shape in the BR mutants and the similarity between BR mutants and tubulin mutants imply that microtubule arrangement or dynamics might be affected in the BR mutants. To clarify this, we examined the microtubule arrangement in BR mutants expressing GFP–tubulin. The *TUBULIN* gene expression levels were detected by real-time PCR (see Supplementary Fig. S2A) and tubulin protein levels were detected by western blotting with anti-GFP antibody (Supplementary Fig. S2B). As shown in Fig. 2, the microtubule organization was altered in the BR mutants. The Microtubule alignment was quantified according to Gomez *et al.* (2016) (Table 2). MTSD is the degree of deviation from the mean direction of the tubulin signal and reduces as microtubules become aligned with each other (Gomez *et al.*, 2016). From Fig. 2A and Table 2, in wild-type mature pavement cells, the microtubules formed a randomly oriented array and were densely localized around the neck region, but in the BR mutants *det2*, *bri1-5*, *bes1-D*, *bin2-1*, and *bin2-3 bil1 bil2*, the microtubules were organized in nearly parallel arrays, with fewer microtubules localized around the neck than in wild-type (Fig. 2B–F; Table 2). These observations indicate that defects in BR biosynthesis and signaling result in an abnormal arrangement of microtubules.

**Table 2.** Microtubule organization of cotyledon pavement cells of BR mutants

	Wild-type	*bri1-5*	*det2*	*bin2-1*	*bin2-3 bil1 bil2*	*bes1-D*	eBL- treated wild-type	BRZ- treated wild-type	Bikinin-treated wild-type	Bikinin-treated *bin2-1*
Random orientation	87%	14.9%****	13.4%****	11.3%****	16.4%****	17.2%****	6.7%****	5.4%****	6.3%****	9.4%****
Paralleled orientation	13%	85.1%****	86.6%****	88.7%****	83.6%****	82.8%****	93.3%****	94.6%****	93.7%****	91.6%****
MTSD (degrees)	52.83 ± 0.76	14.9 ± 4.2****	25.9 ± 2.8****	17.7 ± 3.3****	23.2 ± 1.9****	24.6 ± 2.8****	24.0 ± 7.8****	14.7 ± 0.1****	34.3 ± 1.4****	16.9 ± 3****

MTSD is the degree of deviation from the mean direction of tubulin signal (Gomez *et al.* 2016). *n*>47 cells for each data point. *t*-Test: ****P<0.0001.

### Inhibition of BIN2 activity affects the arrangement of microtubules

**Fig. 3.**
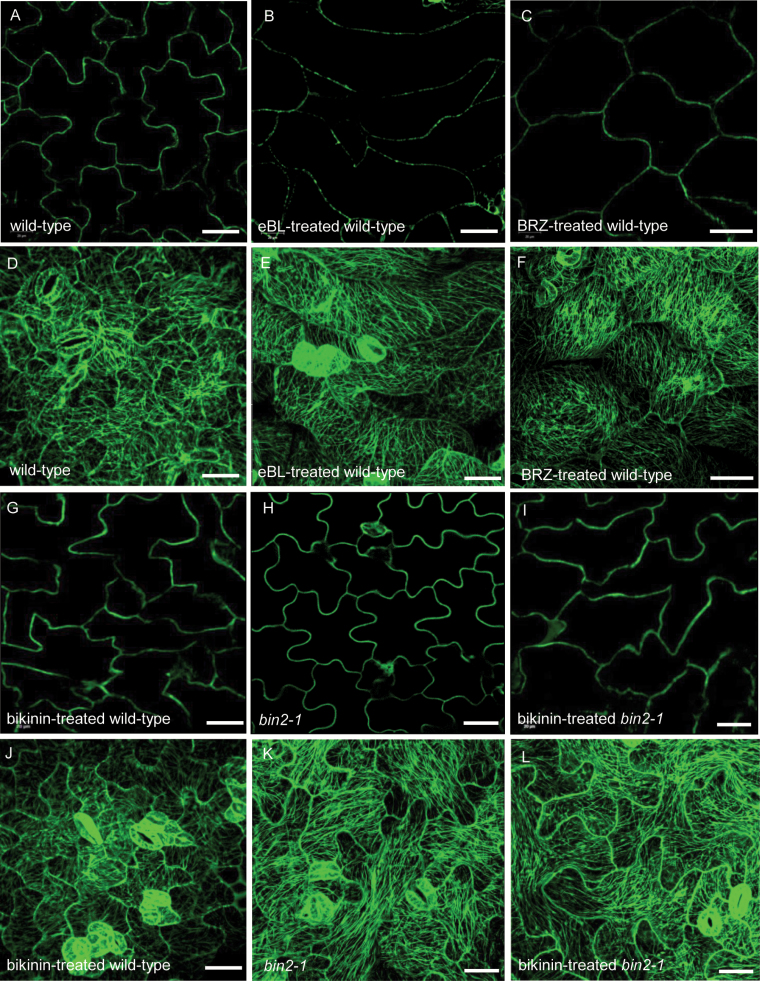
Treatment with eBL, BRZ, and bikinin led to abnormal pavement cell shape and arrangement of microtubules. (A–C, G–I) Pavement cell shape of wild-type (A), eBL-treated wild-type (B), BRZ-treated wild-type (C), bikinin-treated wild-type (G), *bin2-1* (H), and bikinin-treated *bin2-1* (I) cotyledons with a transgene expressing GFP–tubulin in the background for all lines. (D–F, J–L) Cortical microtubules of cotyledon pavement cells in wild-type (D), eBL-treated wild-type (E), BRZ-treated wild-type (F), bikinin-treated wild-type (J), *bin2-1* (K), and bikinin-treated *bin2-1* (L) with a transgene expressing GFP–tubulin in the background for all lines. Bar: 40 μm. (This figure is available in color at *JXB* online.)

**Fig. 4.**
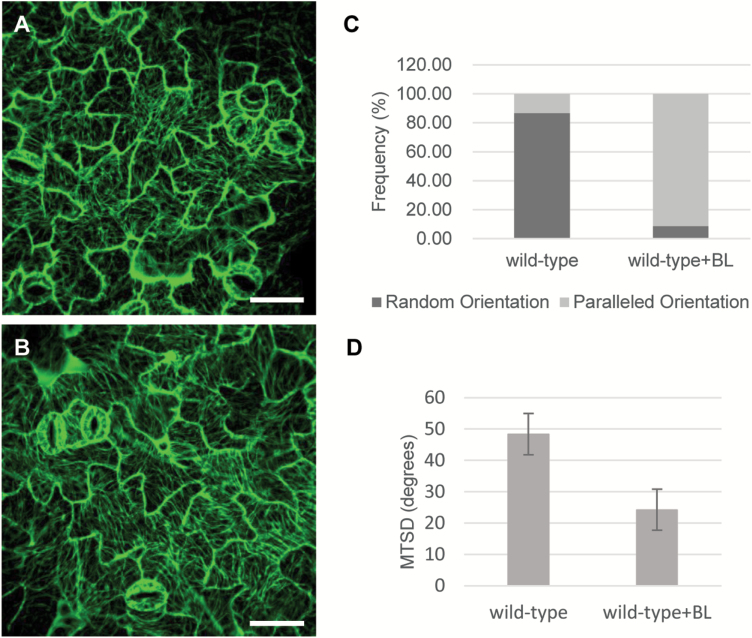
One hour treatment with eBL led to microtubule reorientation. (A) Cortical microtubules of cotyledon pavement cells in wild-type. (B) Cortical microtubules of cotyledon pavement cells in wild-type with eBL treated for 1 h. (C) Frequency of microtubule orientation in pavement cells of wild-type and eBL-treated wild-type. (D) Quantification of MT alignment. Bar: 40 μm. (This figure is available in color at *JXB* online.)

To investigate whether exogenously applied epi-brassinolide (eBL, an active BR) and brassinazole (BRZ, a BR biosynthesis inhibitor) affect microtubule arrangement, we grew wild-type seeds on ½MS medium containing 1 μM eBL or 1 μM BRZ for 7 d. As shown in Fig. 3 and Table 2, when treated with eBL, the wild-type pavement cells changed from jigsaw puzzle shaped to pointed tip shaped (Fig. 3B). By contrast, BRZ-treated pavement cells exhibited a swollen shape (Fig. 3C). Following eBL and BRZ treatment, the microtubule arrangement changed from random to parallel arrays (Fig. 3E, F). Both wild-type and *bin2-1* plants treated with bikinin (an inhibitor of GSK3 kinases) showed similar phenotypes to those of eBL-treated plants (Fig. 3G–L). To detect whether short term treatment of eBL also leads to microtubule reorientation, we treat the wild-type with 10 μM eBL for 1 h. After treatment, the microtubule arrangement in wild-type changed from random to parallel arrays, which is the same as the result of 7 d treatment (Fig. 4A–C). Quantification of microtubule alignment showed that the MTSD changed from 48.26 to 24.16 degrees (Fig. 4D) (Gomez *et al.*, 2016). These results indicate that BR regulates microtubule orientation and affects pavement cell shape directly. The inhibition of BIN2 activity also affects microtubule orientation. Taken together, these results suggest that BR regulates microtubule orientation through its effect on the activity of BIN2.

### BIN2 interacts with tubulin proteins *in vitro* and *in vivo*

**Fig. 5.**
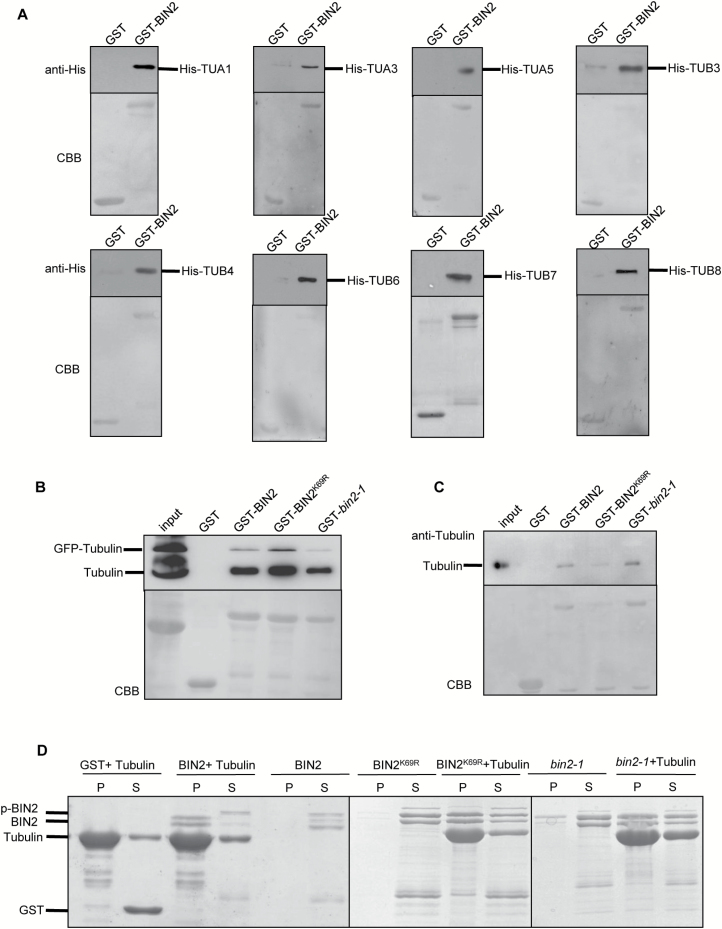
BIN2 interacts with tubulins *in vitro* and semi-*in vivo*. (A) BIN2 directly interacts with tubulins *in vitro* by pull-down assay. Tubulin–His was pulled down by GST–BIN2 immobilized on glutathione resin and analysed by western blotting using anti-His antibody. GST was used as a negative control. GST–BIN2 and tubulin–His were expressed and purified from *E. coli*. The data presented are a western blot (top) and a Coomassie-stained gel (bottom). (B) The interaction between BIN2 and tubulins was detected by a semi-*in vivo* pull-down assay. GST–BIN2 was expressed and purified from *E. coli*. Protein extracts from plants overexpressing tubulin–GFP were used for pull-down assays. Tubulin–GFP and tubulin were pulled down by GST–BIN2, GST–BIN2^K69R^ and GST–bin2-1 immobilized on glutathione resin and analysed by western blotting using anti-α-tubulin antibody. GST was used as a negative control. The data presented are a western blot (top) and a Coomassie-stained gel (bottom). (C) The interaction between BIN2 and tubulins was tested by semi-*in vivo* pull-down assays. Tubulins were pulled down by GST–BIN2, GST–BIN2^K69R^, and GST–bin2-1 immobilized on glutathione resin and analysed by western blotting using anti-tubulin antibody. GST was used as a negative control. GST–BIN2 was expressed and purified from *E. coli* and tubulin was purified from porcine brain. The data presented are a western blot (top) and a Coomassie-stained gel (bottom). (D) The interaction between BIN2 and microtubules was detected by co-sedimentation assays. Recombinant GST–BIN2, GST–BIN2^K69R^, and GST–bin2-1 proteins were co-sedimented with 5 mM Taxol microtubules. Recombinant proteins appeared mainly in the supernatant (S) after centrifugation in the absence of microtubules and co-sedimented with microtubules into the pellets (P) in the presence of microtubules. GST was used as a negative control. (This figure is available in color at *JXB* online.)

To identify proteins that interact with BIN2, we performed LC-MS/MS analysis of proteins that immunoprecipitated with BIN2, using extracts from p*35S*-BIN2::FLAG transgenic plants. The results indicated BIN2 may interact with tubulin proteins. To confirm that BIN2 interacts with tubulin proteins, we conducted pull-down assays. To that end, we cloned the full-length coding sequence of *BIN2* in the pGEX-4T vector and *TUBULIN* genes in the pET28a vector and separately transformed these two constructs into *E. coli*. GST pull-down assays indicated that BIN2 directly interacts with TUA1, TUA3, TUA5, TUB3, TUB4, TUB6, TUB7, and TUB8 *in vitro* (Fig. 5A).

To investigate whether BIN2 interacts with tubulin proteins *in vivo*, we extracted tubulin proteins from plants overexpressing tubulin–GFP, finding that BIN2 interacted with these proteins (Fig. 5B). Because tubulin proteins are dimers comprising α-tubulin and β-tubulin, we purified dimerized tubulin proteins from porcine brain (Fig. 5C) and found that BIN2 interacted with these dimers.

To investigate whether the kinase activity of BIN2 is important for its binding to tubulin proteins, we performed a semi-*in vivo* pull-down assay and found that BIN2–GST, the kinase-dead form BIN2^K69R^, and activated bin2-1 all interacted with tubulin proteins extracted from plants overexpressing tubulin–GFP (Fig. 5B) and with tubulin proteins purified from porcine brain (Fig. 5C).

The expression patterns of α-tubulin, *TUA1*, *TUA3*, and *TUA5*, and β-tubulin, *TUB3*, *TUB4*, *TUB6*, *TUB7*, and *TUB8*, are similar to that of *BIN2* at various stages of development (see Supplementary Fig. S3A–F). This also indicated BIN2 interacted with tubulin proteins in various organ to modulate development.

### BIN2 binds microtubules *in vitro*

Although we determined that BIN2 could bind tubulin proteins, we lacked evidence that it directly affects microtubules. Therefore, we performed a high-speed co-sedimentation assay to investigate whether BIN2 binds directly to microtubules. The full-length coding sequences of *BIN2*, *BIN2*^*K69R*^, and *bin2-1* were cloned separately into the pGEX-4T vector. Recombinant proteins GST–BIN2, GST–BIN2^K69R^, and GST–bin2-1 were incubated individually with pre-formed Taxol-stabilized microtubules (5 mM) at room temperature for 20 min. After centrifugation at 100000 *g*, the supernatants and pellets were subjected to SDS-PAGE. Before the addition of microtubules, BIN2, BIN2^K69R^, and bin2-1 were only present in the supernatant (Fig. 5D). After incubation and centrifugation, BIN2, BIN2^K69R^, and bin2-1 were detected in the pellet, indicating that all three proteins directly bound to and co-sedimented with microtubules (Fig. 5D).

### The *bin2-3 bil1 bil2* triple mutant is more sensitive to the microtubule-disrupting drug oryzalin than wild-type and *bin2-1*

**Fig. 6.**
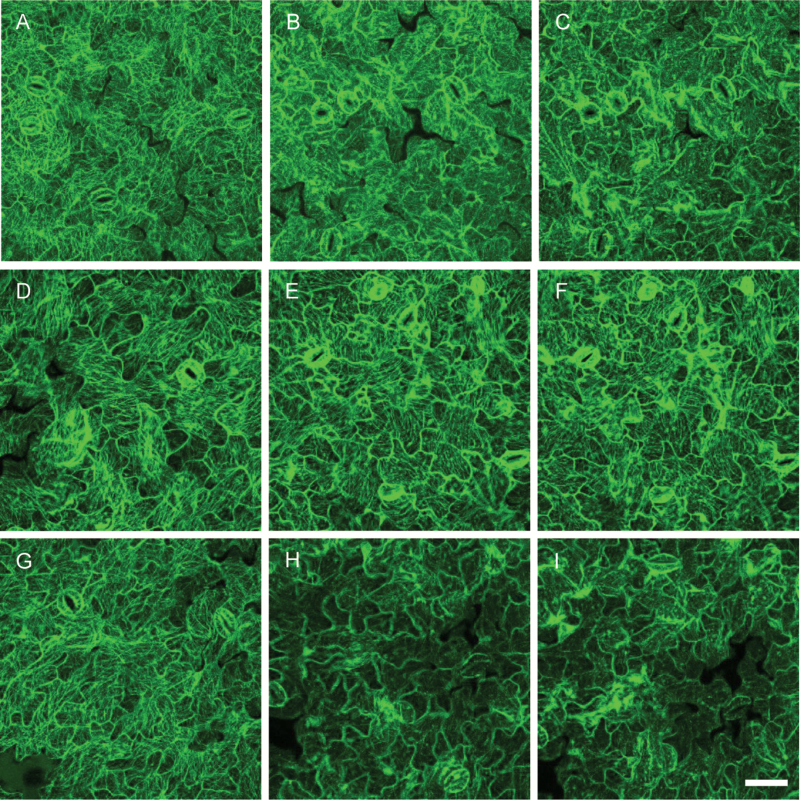
Microtubules are hypersensitive to treatment with oryzalin in *bin2-3 bil1 bil2* cells but more resistant to oryzalin in *bin2-1* cells. Cortical microtubules were observed in the cotyledon pavement cells of the wild-type (A–C), *bin2-1* (D–F), and *bin2-3 bil1 bil2* (G–I) plants after treatment with 0 (A, D, G) or 15 μM oryzalin (B, E, H) for 3 min or 15 μM oryzalin for 10 min (C, F, I). Bar in (I): 50 μm. (This figure is available in color at *JXB* online.)

Both the *bin2-1* and the *bin2-3 bil1 bil2* mutants exhibited an abnormal microtubule arrangement, prompting us to investigate the relationship between this phenotype and the stability of cortical microtubules. To investigate microtubule stability, we examined the microtubule arrangement in *bin2-1* and *bin2-3 bil1 bil2* plants expressing GFP–tubulin and treated with the microtubule-disrupting drug oryzalin. As shown in Fig. 6A, D, G, before treatment the microtubules were arranged in a more parallel orientation in *bin2-1* (Fig. 6D) and *bin2-3 bil1 bil2* (Fig. 6G) than in wild-type (Fig. 6A). After 3 min of treatment with 15 μM oryzalin, the number of cortical microtubules in pavement cells differed between wild-type, *bin2-1*, and *bin2-3 bil1 bil2* plants. The microtubules were disrupted in *bin2-3 bil1 bil2* pavement cells (Fig. 6H) and weakly disrupted in wild-type pavement cells (Fig. 6B), while the microtubules in *bin2-1* pavement cells were almost normal (Fig. 6E). When we increased the treatment time to 10 min, the microtubules in *bin2-3 bil1 bil2* (Fig. 6I) and wild-type pavement cells were almost completely disrupted (Fig. 6C), while microtubules in *bin2-1* were almost normal (Fig. 6F). These results indicate that the binding of BIN2 has a stabilizing effect on microtubules.

## Discussion

In this study, we provided several lines of evidence showing that BR plays important roles in regulating microtubule arrangement and stability via BIN2. First, pavement cell shape was dramatically altered in BR-defective mutants and in wild-type plants grown on ½MS medium containing eBL, bikinin, or BRZ for 7 d. Seven-day treatment and 1 h treatment of eBL led to microtubule reorientation demonstrating that GSK3-like kinase, BIN2, or its upstream components are involved in this regulation. Second, BIN2 directly interacted with tubulin proteins, suggesting that it directly regulates microtubule organization. Third, BIN2 binds microtubules, suggesting that it directly regulates microtubule stability and arrangement. Finally, the *bin2-1* mutant was insensitive to the microtubule-disrupting drug oryzalin whereas the *bin2-3 bil1 bil2* triple mutant was hypersensitive to oryzalin, indicating that BIN2 regulates pavement cell growth and leaf development by stabilizing microtubules.

Microtubules are formed by the polymerization of α-tubulin and β-tubulin dimers. Polymerization-biased dynamic instability at the plus end and slow depolymerization at the minus end contribute to cortical microtubule dynamics. Dynamic microtubules become organized into cortical microtubule arrays. The suppression of cortical microtubule dynamics and organization results in abnormal cell morphogenesis. Interference with the microtubule cytoskeleton results in pavement cells with defects in lobe formation. In the current study, we found that BR biosynthesis-defective mutants had round leaves, as well as smaller and fewer lobes in their pavement cells compared with wild-type, while BR overexpression lines showed right-handed helical growth in leaves and spindle-like pavement cells. The microtubule-related mutants such as *sav2* had round, small rosette leaves and round and wide pavement cells, which is similar to the phenotype of BR-defective mutants (see Supplementary Fig. S1). The similarity between BR mutants and microtubule-related mutants suggests that BR might mediate pavement cell growth through its effect on cortical microtubules. We also found that BIN2 interacted with both α-tubulin and β-tubulin (Fig. 5). Thus, it is highly likely that the binding of tubulins by BIN2 affects tubulin dimerization and microtubule organization.

We also performed an *in vitro* kinase assay to investigate whether BIN2 can phosphorylate tubulin proteins (see Supplementary Fig. S4). GST–BIN2 and GST– BIN2^K69R^ were incubated separately with dimerized tubulins extracted from porcine brain for 1 h at 37 °C. Autoradiography showed that BIN2 could phosphorylate these proteins, but the kinase-dead form of BIN2 (BIN2^K69R^) could not. Therefore, while both BIN2 and BIN2^K69R^ bind tubulin proteins, only BIN2 can phosphorylate these proteins. Although we did not determine which tubulin protein is phosphorylated by BIN2 and which phosphorylation site is responsible for the phosphorylation and the resulting phenotype, our findings suggest that the interaction and phosphorylation of these proteins affect microtubule organization and stability.

Using a co-sedimentation assay, we confirmed the binding between BIN2 and microtubules. To investigate the binding mechanism *in vivo*, we treated *bin2-1* and *bin2-3 bil1 bil2* triple mutant plants with the microtubule-disrupting drug oryzalin. The *bin2-3 bil1 bil2* mutant showed hypersensitivity to oryzalin treatment, while *bin2-1* was insensitive. Many microtubule-binding proteins in plants have been reported, such as MAP65 (Mao *et al.*, 2005), MAP18 (Wang *et al.*, 2007), and MDP25 (Li *et al.*, 2011). Microtubule-binding proteins are considered to be microtubule stabilizers or destabilizers depending on their effect on microtubule stability. Among these, MAP65 is a microtubule stabilizer, whereas MAP18 and MDP25 are microtubule destabilizers. A *MAP18* overexpression line (Wang *et al.*, 2007), in which cortical microtubules were destabilized, had a similar response to oryzalin treatment to that of the *bin2-3 bil1 bil2* triple mutant, indicating that BIN2 is also a microtubule stabilizer. BR likely mediates pavement cell growth through its effect on the interaction with tubulin proteins and the stabilization of microtubules by BIN2.

*BIN2* is expressed in various organs, suggesting that BIN2 plays a general role in regulating microtubule dynamics. Treatment with the microtubule-disrupting drug oryzalin leads to left-skewing root growth. The microtubule-binding protein mutant *spr1* shows a right-skewing root growth phenotype; the direction of skewing is reversed by treatment with 3 µM microtubule depolymerizing drug propyzamide (Furutani *et al.*, 2000; Sedbrook *et al.*, 2004). The α-tubulin mutant *lefty* and a *MAP18* overexpression line both exhibit left-skewing root growth due to inhibited tubulin polymerization. Therefore, the stabilization and disruption of cortical microtubules both lead to root growth defects. To investigate the role of BIN2 in root growth, we compared the roots of *bin2-1* and *bin2-3 bil1 bil2* with those of wild-type. When treated with 0.1 µM oryzalin, *bin2-1* roots were significantly shorter than those of wild-type and skewed to the right, whereas *bin2-3 bil1 bil2* roots were longer than wild-type and skewed to the left (see Supplementary Fig. S5). These observations indicate that BIN2 is a microtubule-stabilizing factor that plays an important role in regulating plant growth and development.

BR regulates both hypocotyl elongation and pavement cell development. Hypocotyl growth is polarized growth, whereas pavement cell development is a much more intricate process involving a combination of localized tip growth and diffuse growth to produce interlocking cells with complex shapes. Pavement cell morphogenesis is controlled by the countersignaling of two pathways with opposite effects (Fu *et al.*, 2005). In addition to BR, auxin regulates plant growth in various ways depending on the developmental stage and plant organ (Nick *et al.*, 1992). In roots and hypocotyls, auxin treatment alters the arrangement of microtubules towards a longitudinal direction (Chen *et al.*, 2014), but in shoots, auxin treatment leads to a transverse microtubule orientation (Sassi *et al.*, 2014). Therefore, BR may regulate microtubule stability differently in pavement cells than in hypocotyls. BZR1, a transcription factor in the BR signal pathway, can target and up-regulate *MDP40* to mediate hypocotyl cell elongation by influencing the orientation and stability of cortical microtubules (Wang *et al.*, 2012). In the current study, we found that in pavement cells, BR regulates microtubule dynamics through direct binding to BIN2. We also examined the microtubule arrangements in hypocotyls of *bin2-1* and *bin2-3 bil1 bil2* triple mutants and found the microtubules in *bin2-1* and *bin2-3 bil1 bil2* triple mutants were oblique to the long expansion axis of the cell while in wild-type the microtubules were transversely reoriented to the long expansion axis of the cell in the upper regions of hypocotyls (see Supplementary Fig. S6). While BIN2 can phosphorylate BZR1 directly, the abnormality of microtubule arrangements in *bin2-1* and *bin2-3 bil1 bil2* triple mutants may be caused by the abnormal BZR1. Although the mechanism uncovered in the current study differs from that described by Wang *et al.* (2012), the effect of BR on microtubules is the same. Both studies indicated that BR regulates plant growth by destabilizing microtubules.

Auxin modulates the interdigitated growth of leaf pavement cells and cell expansion through an ABP1-related pathway that activates ROP2 and ROP6 (Xu *et al.*, 2010). Auxin activates ROP6 to modulate the association of RIC1 with microtubules and ROP2 to activate RIC4, promoting the assembly of cortical actin microfilaments required for localized outgrowth (Fu *et al.*, 2005). Gibberellic acid (GA) also affects cortical microtubule orientation. The application of GA induces a transverse cortical microtubule array organization through the interaction between DELLA and the prefoldin proteins PFD3 and PFD5 (Locascio *et al.*, 2013; Vineyard *et al.*, 2013). DELLAs interact with PFD3 and PFD5, causing them to be retained in the nucleus to inhibit α-tubulin and β-tubulin heterodimerization. When GA levels are high, degraded DELLAs release PFD3 and PFD5 into the cytoplasm to promote tubulin dimerization (Locascio *et al.*, 2013). Ethylene inhibits etiolated hypocotyl elongation through the activity of EIN3, which directly targets and up-regulates the expression of the microtubule-stabilizing protein WDL5 (Sun *et al.*, 2015). Most phytohormones regulate plant growth through their effects on microtubules, suggesting that there is crosstalk among hormonal signaling pathways. The relationship between these phytohormones and how they regulate the cytoskeleton represents an exciting and fertile area of research. Our finding that BR regulates pavement cell formation through its effect on the binding and stabilization of microtubules by BIN2 contributes to our understanding of this important topic.

## Supplementary data

Supplementary data are available at JXB online.

Fig. S1. The *tub4* mutant shows an abnormal rosette leaf phenotype and pavement cell shape.

Fig. S2. The relative expression levels of *TUBULIN* gene and protein levels of tubulin in BR mutants.

Fig. S3. Expression patterns of *TUBULIN* genes.

Fig. S4. BIN2 phosphorylates tubulins *in vitro*.

Fig. S5. The *bin2-1* and *bin2-3 bil1 bil2* mutants exhibit a skewed-root phenotype when treated with 0.1 μM oryzalin.

Fig. S6. The cortical microtubule array is altered in hypocotyl epidermal cells of *bin2-1* and *bin2-3 bil1 bil2* mutants.

Table S1. Primers used for real-time PCR.

Supplementary Figures and Table
