# Supplementary material for: Brassinosteroids regulate pavement cell growth by mediating BIN2-induced microtubule stabilization
Source: J Exp Bot. 2018 Jan 10;69(5):1037–49. doi: 10.1093/jxb/erx467 (PMC6018924; doi:10.1093/jxb/erx467)
Supplement: Supplementary Figures and Table [file erx467_suppl_supplementary_figures_and_table.pdf]

Brassinosteroids regulate pavement cell growth by mediating BIN2-induced microtubule stabilization

Xiaolei Liu[a,b,1], Qin Yang[b], Yuan Wang[b,e], Linhai Wang[c], Ying Fu[c], Xuelu Wang[d]

[a]Shanghai Center for Plant Stress Biology, Chinese Academy of Sciences, Shanghai 201602, China

[b]State Key Laboratory of Genetic Engineering and Institute of Plant Biology, School of Life Sciences, Fudan University, Shanghai 200433, China;

[c]State Key Laboratory of Plant Physiology and Biochemistry, Department of Plant Sciences, College of Biological Sciences, China Agricultural University, Beijing 100193, China;

[d]College of Life Science and Technology, Huazhong Agricultural University, Wuhan 430070, China;

[e]Department of Botany and Plant Science, University of California Riverside, Riverside 92507, United States;

[1]To whom correspondence should be addressed. E-mail: liuxiaolei@sibs.ac.cn

Figure S1

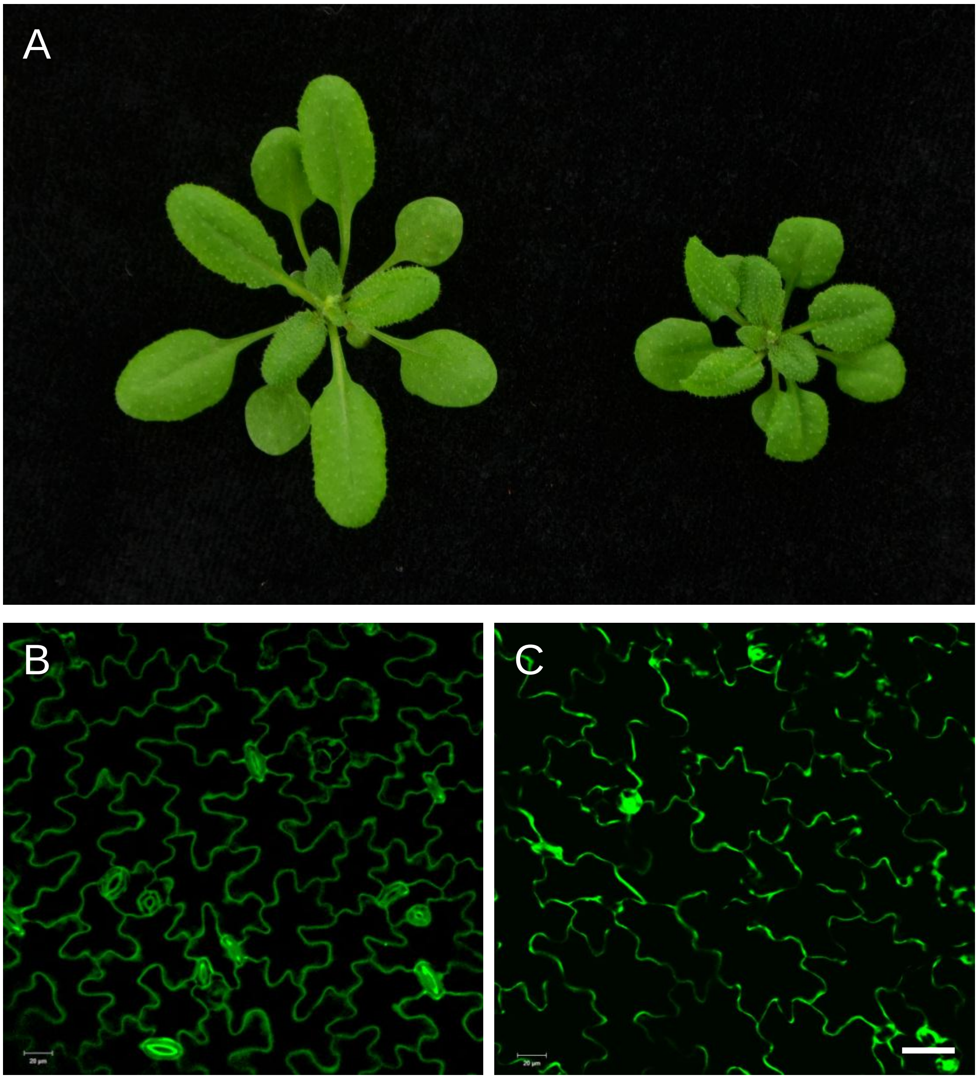

Figure S1. The *tub4* mutant shows an abnormal rosette leaf phenotype and pavement cell shape.

(A) Phenotype of the *tub4* mutant.

(B) Pavement cell shape of wild-type (B) and *tub4* mutant (C) cotyledons stained by propidium iodide.

Bar in (C) = 40μm

Figure S2

A

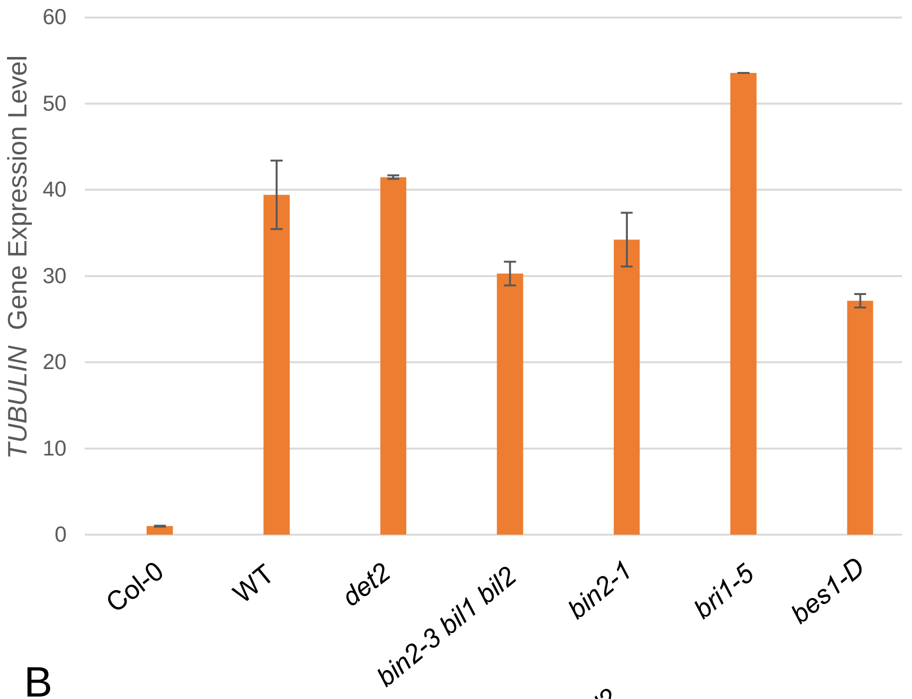

60
50
40
30
20
10
0
TUBULIN Gene Expression Level
Col-0
WT
det2
bin2-3 bil1 bil2
bin2-1
bri1-5
bes1-D

B

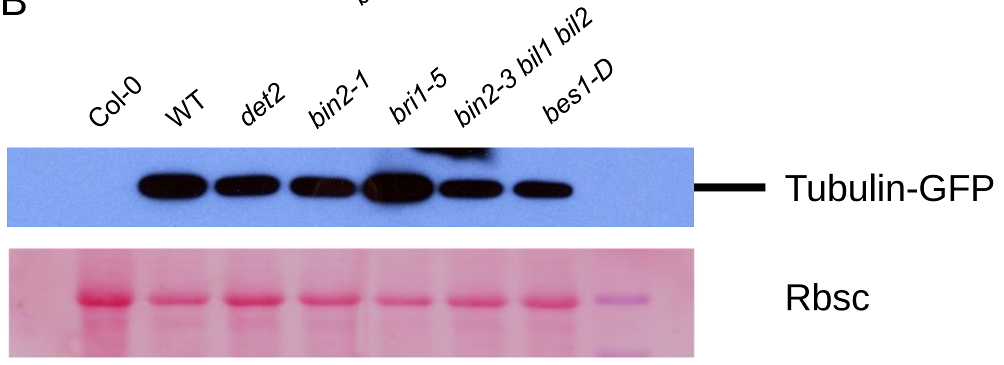

Col-0
WT
det2
bin2-1
bri1-5
bin2-3 bil1 bil2
bes1-D
Tubulin-GFP
Rbsc

- Figure S2. The relative expression levels of TUBULIN gene and protein levels of tubulin in BR mutants.

(A) The relative expression levels of the TUBULIN in wild-type, *bri1-5*, *det2*, *bin2-1*, *bin2-3 bil1 bil2*, *bes1-D* with GFP-tubulin background were detected by realtime PCR.

(B) The tubulin protein levels in Col-0, wild-type, *bri1-5*, *det2*, *bin2-1*, *bin2-3 bil1 bil2*, *bes1-D* with GFP-tubulin background were detected by western blotting with anti-GFP antibody.

Figure S3

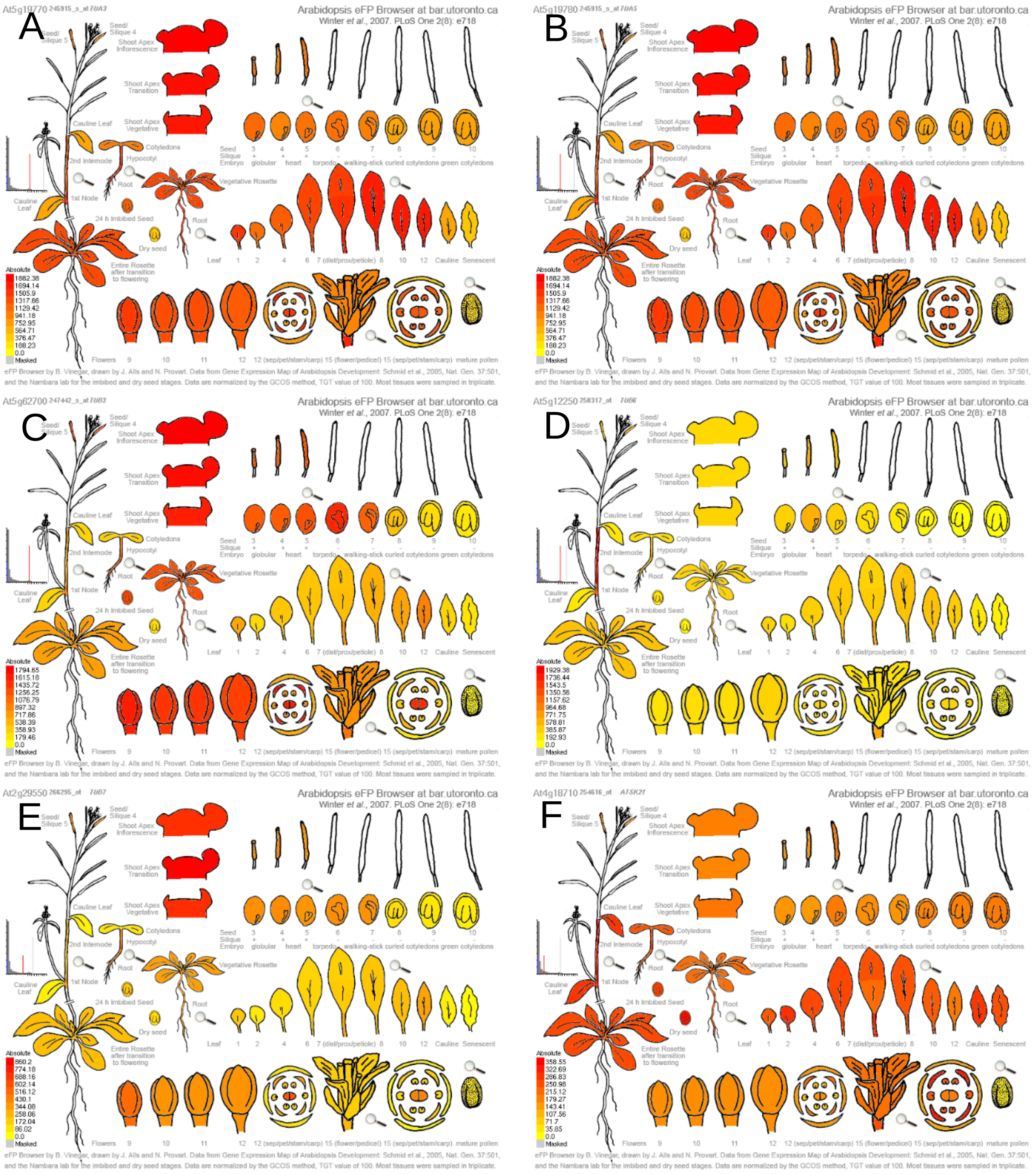
A
At5g19770 245915_s_at TUA3
Arabidopsis eFP Browser at bar.utoronto.ca
Winter et al., 2007. PLoS One 2(8): e718
Absolute 1882.38 1694.14 1505.9 1317.66 1129.42 941.18 752.95 564.71 376.47 188.23 0.0 Masked
B
At5g19780 245915_s_at TUA5
Absolute 1882.38 1694.14 1505.9 1317.66 1129.42 941.18 752.95 564.71 376.47 188.23 0.0 Masked
C
At5g62700 247442_s_at TUB3
Absolute 1794.65 1615.18 1435.72 1256.25 1076.79 897.32 717.86 538.39 358.93 179.46 0.0 Masked
D
At5g12250 250317_at TUB6
Absolute 1929.38 1736.44 1543.5 1350.56 1157.62 964.68 771.75 578.81 385.87 192.93 0.0 Masked
E
At2g29550 266295_at TUB7
Absolute 860.2 774.18 688.16 602.14 516.12 430.1 344.08 258.06 172.04 86.02 0.0 Masked
F
At4g18710 254616_at ATSK21
Absolute 358.55 322.69 286.83 250.98 215.12 179.27 143.41 107.56 71.7 35.85 0.0 Masked
Seed/ Silique 4
Seed/ Silique 5
Shoot Apex Inflorescence
Shoot Apex Transition
Shoot Apex Vegetative
Cauline Leaf
2nd Internode
1st Node
Cotyledons
Hypocotyl
Root
Vegetative Rosette
24 h Imbibed Seed
Dry seed
Entire Rosette after transition to flowering
Seed Silique Embryo 3 4 5 6 7 8 9 10 globular heart torpedo walking-stick curled cotyledons green cotyledons
Leaf 1 2 4 6 7 (dist/prox/petiole) 8 10 12 Cauline Senescent
Flowers 9 10 11 12 12 (sep/pet/stam/carp) 15 (flower/pedicel) 15 (sep/pet/stam/carp) mature pollen
eFP Browser by B. Vinegar, drawn by J. Alls and N. Provart. Data from Gene Expression Map of Arabidopsis Development: Schmid et al., 2005, Nat. Gen. 37:501, and the Nambara lab for the imbibed and dry seed stages. Data are normalized by the GCOS method, TGT value of 100. Most tissues were sampled in triplicate.

Figure S3. Expression of *TUBULIN* genes.

(A) to (F) *TUA3* (A), *TUA5* (B), *TUB3* (C), *TUB6* (D), *TUB7* (E), *BIN2* (F) are expressed extensively in different development stages. Microarray data are from AtGenExpress.

Figure S4

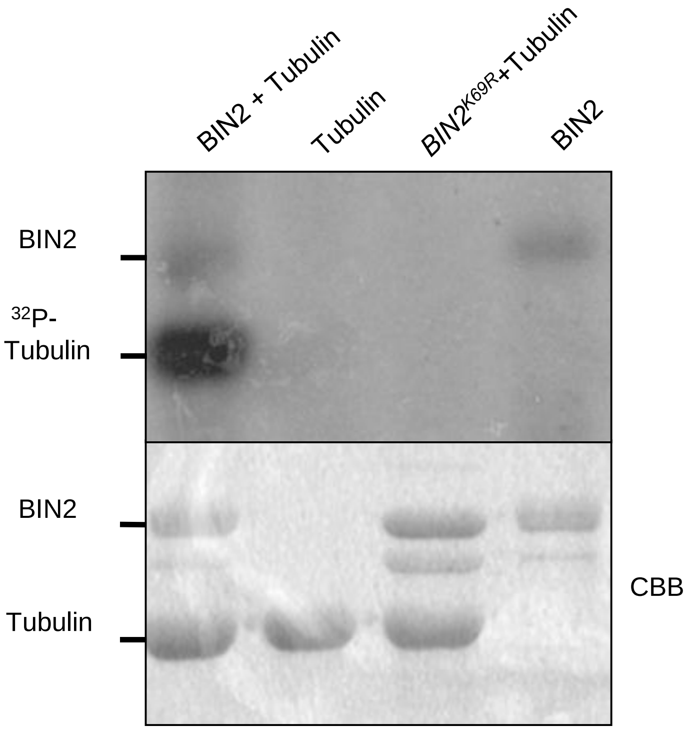
BIN2 + Tubulin
Tubulin
$BIN2^{K69R}$+Tubulin
BIN2
BIN2
^{32}P-
Tubulin
BIN2
Tubulin
CBB

Figure S4. BIN2 phosphorylates tubulins in vitro.

Recombinant GST-BIN2 was incubated with tubulins from porcine brain. GST-BIN2^{K69R} was used as a negative control. Phosphorylated tubulins were visualized by autoradiography after gel electrophoresis and Coomassie Brilliant Blue (CBB) was used to visualize total proteins.

Figure S5

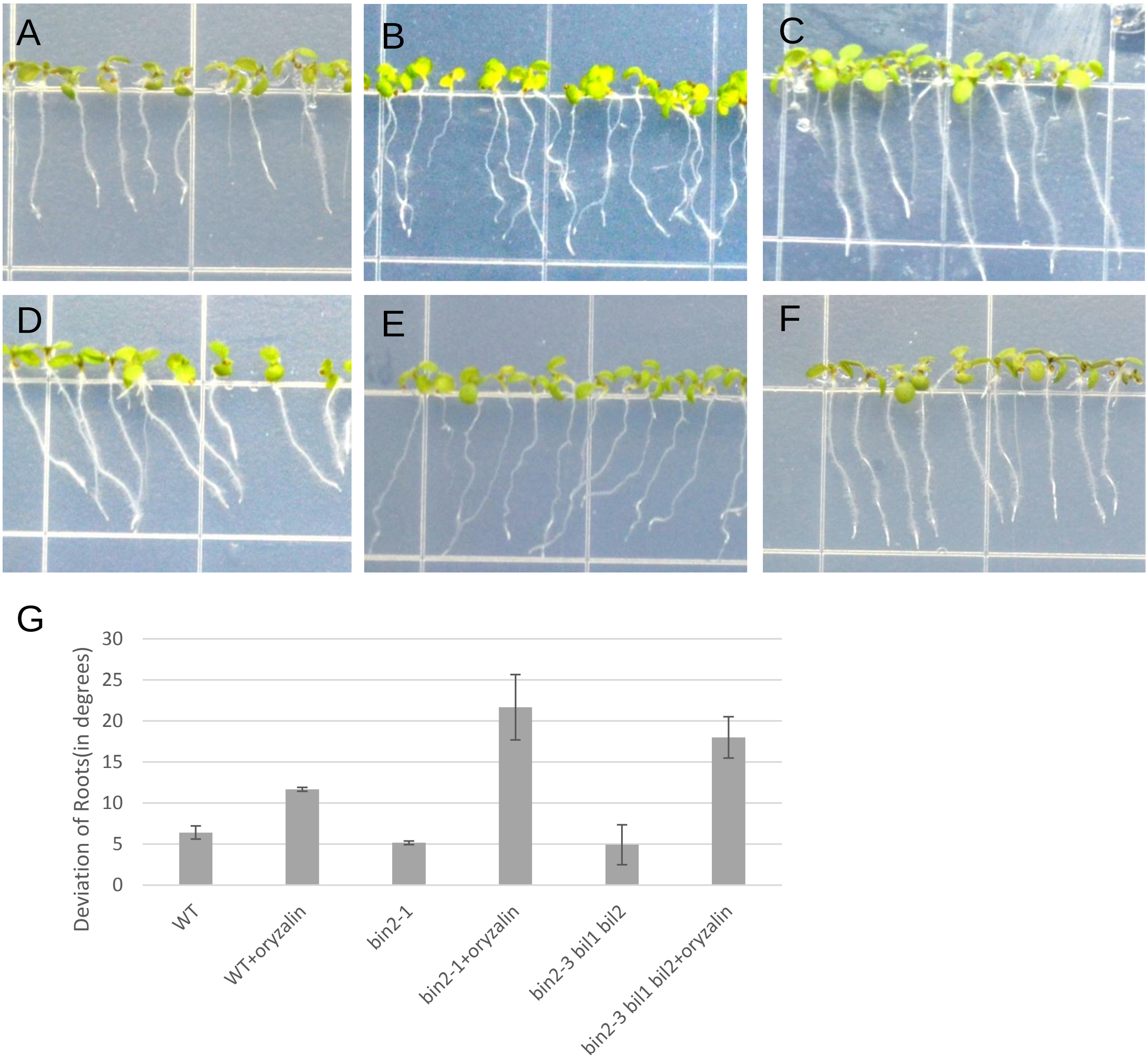
A
B
C
D
E
F
G
Deviation of Roots(in degrees)
30
25
20
15
10
5
0
WT
WT+oryzalin
bin2-1
bin2-1+oryzalin
bin2-3 bil1 bil2
bin2-3 bil1 bil2+oryzalin

Figure S5. The *bin2-1* and *bin2-3 bil1 bil2* triple mutants exhibit a skewed-root phenotype when treated with 0.1 μM oryzalin.

A. Seven-day-old *bin2-1* seedlings were grown vertically in the presence of light. Phenotype of *bin2-1* roots.

B. Seven-day-old *bin2-3 bil1 bil2* seedlings were grown vertically in the presence of light. Phenotype of *bin2-3 bil1 bil2* triple mutant roots.

C. Seven-day-old wild-type seedlings were grown vertically in the presence of light. Phenotype of wild-type roots.

D. Seven-day-old *bin2-1* seedlings were grown vertically in the presence of 0.1 μM oryzalin. Phenotype of *bin2-1* roots.

E. Seven-day-old *bin2-3 bil1 bil2* seedlings were grown vertically in the presence of 0.1 μM oryzalin. Phenotype of *bin2-3 bil1 bil2* triple mutant roots.

F. Seven-day-old wild-type seedlings were grown vertically in the presence of 0.1 μM oryzalin. Phenotype of wild-type roots.

G. Quantification of the deviation of mutant roots.

Figure S6

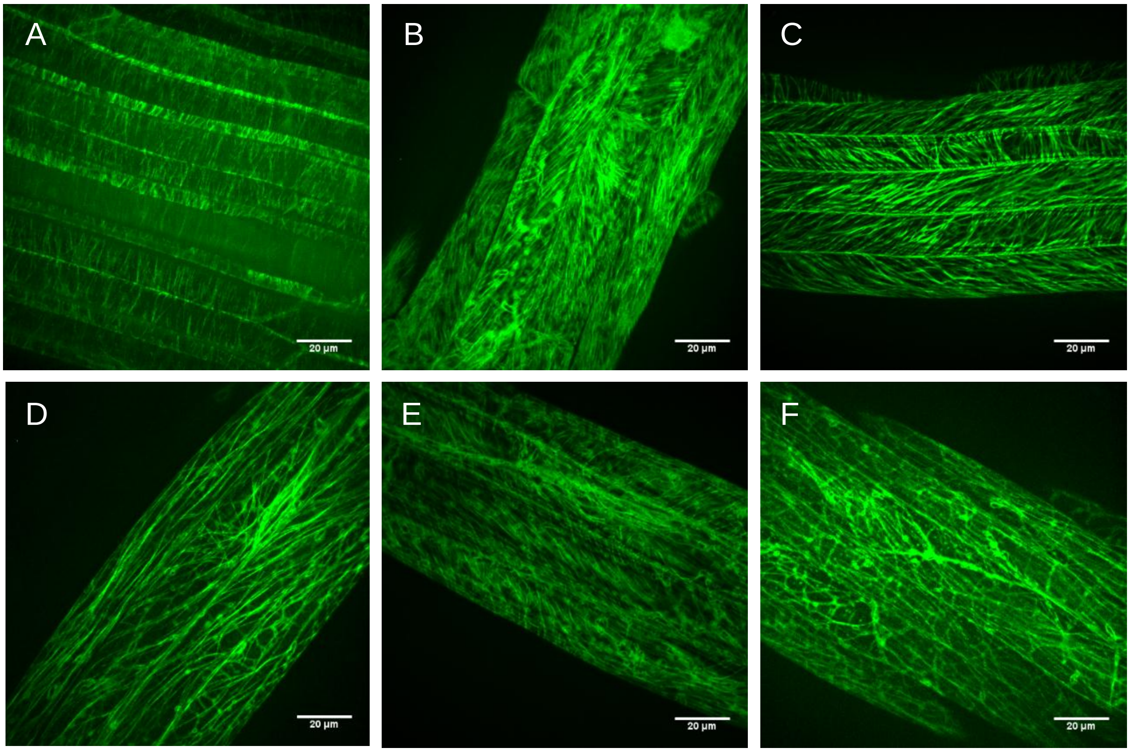

Figure S6. The cortical microtubule array is altered in hypocotyl epidermal cells of *bin2-1* and *bin2-3 bil1 bil2* mutants.

(A) to (C) Cortical microtubules of hypocotyls epidermal cells of wild-type(A), *bin2-1* (B), *bin2-3 bil1 bil2* (C) with GFP-tubulin background in upper hypocotyl cells.

(D) to (F) Cortical microtubules of hypocotyls epidermal cells of of wild-type(D), *bin2-1* (E), *bin2-3 bil1 bil2* (F) with GFP-tubulin background in basal hypocotyl cells.

Table S1. Primers used for Realtime PCR.

Primer name	Primer sequence(5’-3’)
UBOX-F	TCTTCTTCTGCTACATCTACTCTC
UBOX-R	AGTGTGTGAACCCGTGAAC
TUBULIN-F	CATGGCATTCAGCCTGATGG
TUBULIN-R	CTTTCTGATCCGGTCCAAGC
